# A novel prothrombin time method to measure all non-vitamin K-dependent oral anticoagulants (NOACs)

**DOI:** 10.1080/03009734.2017.1370040

**Published:** 2017-09-11

**Authors:** Tomas L. Lindahl, Kerstin Arbring, Maria Wallstedt, Mats Rånby

**Affiliations:** a Department of Clinical Chemistry, Linköping University, Linköping, Sweden;; b Department of Clinical and Experimental Medicine, Linköping University, Linköping, Sweden;; c Department of Acute Internal Medicine, Linköping University, Linköping, Sweden;; d Department of Medical and Health Sciences, Linköping University, Linköping, Sweden;; e Zafena AB, Borensberg, Sweden

**Keywords:** Anticoagulant, apixaban, dabigatran, prothrombin time, rivaroxaban

## Abstract

**Background:**

There is a clinical need for point-of-care (POC) methods for non-vitamin K-dependent oral anticoagulants (NOACs). We modified a routine POC procedure: Zafena’s Simple Simon™ PT-INR, a room-temperature, wet-chemistry prothrombin time method of the Owren-type.

**Methods:**

To either increase or decrease NOAC interference, two assay variants were devised by replacing the standard 10 µL end-to-end capillary used to add the citrated plasma sample to 200 µL of prothrombin time (PT) reagent by either a 20 µL or a 5 µL capillary. All assay variants were calibrated to show correct PT results in plasma samples from healthy and warfarin-treated persons.

**Results:**

For plasmas spiked with dabigatran, apixaban, or rivaroxaban, the 20 µL variant showed markedly higher PT results than the 5 µL. The effects were even more pronounced at room temperature than at +37 °C. In plasmas from patients treated with NOACs (*n* = 30 for each) there was a strong correlation between the PT results and the concentration of NOACs as determined by the central hospital laboratory. For the 20 µL variant the PT response of linear correlation coefficient averaged 0.90. The PT range was INR 1.1–2.1 for dabigatran and apixaban, and INR 1.1–5.0 for rivaroxaban. Using an INR ratio between the 20 µL and 5 µL variants (PTr20/5) made the NOAC assay more robust and independent of the patient sample INR value in the absence of NOAC. Detection limits were 80 µg/L for apixaban, 60 µg/L for dabigatran, and 20 µg/L for rivaroxaban.

**Conclusions:**

A wet-chemistry POC PT procedure was modified to measure the concentrations of three NOACs using a single reagent.

## Introduction

For more than half a century the vitamin K antagonists (VKAs) were the only oral anticoagulants available to prevent and treat thrombosis in conditions such as atrial fibrillation, venous thromboembolism, and mechanical heart valves. Due to the narrow therapeutic interval, as well as large intra- and inter-individual differences in dose requirements, monitoring of treatment using frequent analysis of prothrombin time is necessary, with adjustment of dosage when needed.

In high-income countries 1% of the population or more are prescribed vitamin K antagonists. Therefore prothrombin time is by far the most common coagulation analysis world-wide. Analysis is performed not only in automated instruments in central laboratories but also to a large extent in wards and primary health care using point-of-care (POC) instruments and even by patients themselves. There are more than 100,000 professional users of POC instruments world-wide, contributing to the good organization of treatment that is the foundation for minimizing complications. The VKAs are efficient, but the main draw-back is increased risk of serious bleeding. In case of bleeding or urgent surgery-specific antidotes, prothrombin complex concentrates are available and POC instruments help, guiding expedited treatment.

In recent years NOACs have been added to the armamentarium of the clinicians. So far one direct thrombin inhibitor, dabigatran, and three direct factor Xa inhibitors, rivaroxaban, edoxaban and apixaban, have been approved for clinical use in Europe. In contrast to VKAs the NOACs are argued by the manufacturers not to require routine monitoring. However, patient exposure to the drugs varies substantially and is affected by e.g. renal function, age, body weight, dosage, and drug interactions. For dabigatran, an increased risk of major bleeding has been shown to correlate to the trough concentration, and the risk of ischemic events was inversely related to trough dabigatran concentrations (1). Maybe selective monitoring of NOAC therapy to optimize effects and safety in certain patient groups would be useful. More important, in many clinical situations knowledge of the concentration of the drug would help in decision-making, for example elective and emergency surgery, bleeding, and thrombosis. At present, there is a specific antidote available only for dabigatran, but such antidotes are in development for factor Xa inhibitors (2–4). The clinical need to know the concentration of NOACs may be urgent at present and will be even more so when more specific antidotes are available. There are several functional assays on the market and more in development, but to the best of our knowledge none intended for point-of-care analysis.

It is a well-known fact that, in most analytical procedures including prothrombin time (PT), analytical interferences increase when the sample dilutions decrease. PT determination at high and low sample dilution may thus be expected to disclose an analytical interference, e.g. the presence of NOAC, as a ratio of PT results. Results of previous studies have indicated that the Owren-type PT (i.e. a combined thromboplastin which allows extensive dilution of sample) (5), with a final sample dilution 1:21, is less sensitive to NOACs, heparins, and lupus anticoagulant (6) than the Quick PT (i.e. plain thromboplastin), with a final sample dilution of 1:3. The Owren PT has in fact been shown to be much less sensitive to NOACs than Quick PT (7–9). Using this knowledge, we modified an existing POC PT procedure aiming at providing readily available, short turn-around time NOAC measurements using one single reagent.

## Materials and methods

### POC instrument, procedure modifications, and comparisons with standard procedures

The PT-INR POC instrument Simple Simon™ (Zafena AB, Borensberg, Sweden) is a room-temperature, wet-chemistry PT of the Owren-type, able to operate on both citrated plasma and whole blood, presenting the result as INR. It is used in a large number of primary health care centers in Sweden and Norway, mainly for monitoring of warfarin treatment. In this method PT is expressed in INR as calibrated against plasmas with known INR by established laboratory procedure (10). Calibration is performed by the manufacturer for each lot of reagent, and the calibration and reagent are stable until expiration date, at least one year.

The Simple Simon™ instrument was used as supplied, and with two modifications: the standard 10 µL end-to-end capillary used to introduce the sample into the 200 µL of PT Owren reagent lot N223M (Zafena AB) was substituted by a 20 µL and a 5 µL capillary. Thus, the modifications featured final sample dilutions of 1:11 and 1:41, respectively, in addition to the original 1:21 dilution.

The Simple Simon™ readers generate a basal PT result expressed in INR, here called INR_B_, which is calculated with respect to the temperature at which it is generated. Calibration is accomplished by adjusting two constants, A and B, which affects the INR displayed by the reader according to Equation (1):
(1)
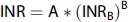



The reader delivered by Zafena for the study had A and B values of 0.813 and 0.960, respectively. With addition of 10 µL of sample to the specified 200 µL of reagent, these constants allowed the Simple Simon equipment of lot N223M to deliver INR values in good agreement with Nordic hospital laboratories as shown by the results obtained for this lot in the external quality assurance schemes of the Norwegian external quality assessment organization NOKLUS (Bergen, Norway) and the Swedish external quality assessment organization EQUALIS (Uppsala, Sweden). To make the readers generate the same INR with 20 µL and 5 µL of sample as with 10 µL, the constants A and B were adjusted to 0.924 and 1.112 for readers working with 20 µL sample, and to 0.691 and 0.822 for readers working with 5 µL of sample, respectively, through calibration using authentic plasma samples from healthy donors (*n* = 30) and patients on warfarin (*n* = 30). The alignment between the results with 20 µL of sample and 10 µL of sample (Supplementary Figure S-1A, available online) and that of 5 µL of sample and 10 µL of sample (Supplementary Figure S-1B, available online) displayed INR independent comparison coefficient of variation (CV) of 6.5% and 5.5%, respectively.

PT was also measured on the automated coagulation instrument ACL Top (Instrumentation Laboratory, Milan, Italy) at +37 °C using 20 µL, 10 µL, or 5 µL plasma and the same lot of PT Owren reagent from Zafena.

### Estimation pf NOAC concentration

To estimate NOAC concentration three INR determinations were performed, one by the 20 µL sample addition, one by 10 µL, and one by the 5 µL sample addition, and the ratio of INR of the 20 µL and 5 µL sample additions (PTr20/5) was calculated to compensate for variation in INR values in between patients in the absence of NOAC. For rivaroxaban, a calibration curve was made on citrated blood, and citrated blood samples from three patients were measured and the ratio calculated in the same way.

### Central laboratory methods for NOAC measurements

The reference method for dabigatran was the clinically used Hemoclot dTT^®^ (Hyphen Biomed, Neuville-sur-Oise, France), and for rivaroxaban and apixaban dedicated anti-FXa methods, all run at the central laboratory of Linköping University Hospital. None of these methods have been adopted for point-of-care instruments so far.

Dabigatran and rivaroxaban were analyzed on ACL Top (IL, Milan, Italy), and apixaban was run on Sysmex CS 2000i (Siemens Healthcare Diagnostics, Marburg, Germany). Calibrator for dabigatran was from Hyphen Biomed, for rivaroxaban from Technoclone (Vienna, Austria), and for apixaban from Stago (Asnières-sur-Seine, France).

The CV at the central laboratory for dabigatran for mean 110 µg/L was 7.5% (*n* = 14), and for mean 316 µg/L CV was 5.9% (*n* = 15); CV for rivaroxaban for mean 59.4 µg/L was 6.0% (*n* = 40), and for mean 330 µg/L it was 2.1% (*n* = 41); CV for apixaban for mean 96.4 µg/L was 4.1% (*n* = 25), and for mean 322 µg/L it was 4.7% (*n* = 26).

Controls were purchased from Aniara (Gothenburg, Sweden), but for apixaban from Technoclone. The central laboratory takes part in the external control scheme from ECAT (Leiden, The Netherlands) for all NOACs.

### Modified PT methods for measuring NOACs on ACL Top™

The same reagent and lot as used on Simple Simon™ was used on ACL Top with sample volumes 5 µL and 20 µL; PT ratio was calculated as above. In addition, PT was measured using the routine PT method with PT reagent from Medirox (Studsvik, Sweden). Analysis was run at +37 °C. The instrument does not allow for other temperature settings.

### NOAC plasma and blood sample material and ethical approval

Samples with various levels of NOAC were of two kinds, either (a) authentic citrated plasmas or whole-blood samples (from patients on NOAC treatment), or (b) plasma from healthy individuals or a plasma pool spiked with stock solution of either dabigatran, rivaroxaban, or apixaban. Plasma was anticoagulated with citrate, 1/10 volume of 0.109 M citrate in the blood collection vacuum tube. The tubes were centrifuged at 2500*g* for 10 min. Plasma samples were frozen and stored in aliquots at –70 °C until analysis. Fresh whole citrated blood samples from patients were analyzed directly after blood sampling. The active drugs were generously provided by the pharmaceutical companies Boehringer Ingelheim, Bayer, and Bristol-Meyer Squibb.

All blood donors and patients have given informed consent. The study has been approved by the local ethics committee (No 2013/269-32).

## Results

### PTr20/5 in spiked plasma

**Figure 1.**
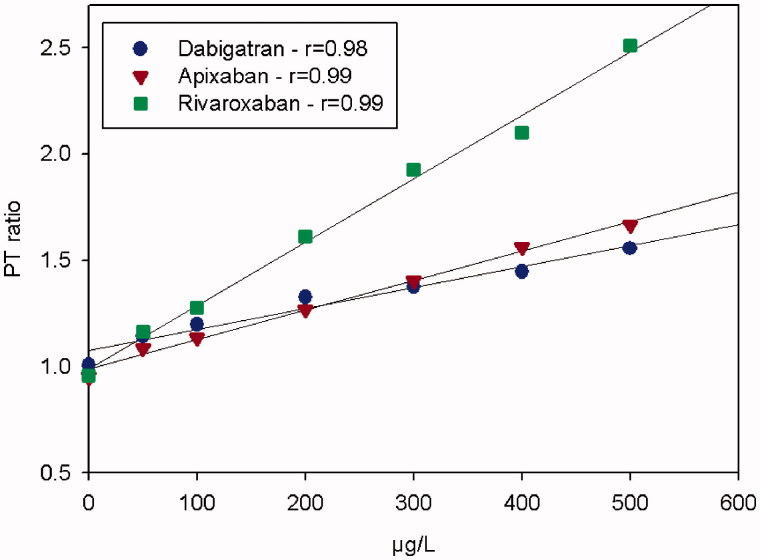
The dose-response characteristics of the PTr method run at room temperature on Simple Simon™ for dabigatran, apixaban, and rivaroxaban in comparison with Hemoclot dTT^®^, our in-house anti-FXa methods, measured on a plasma pool from five healthy donors spiked with the drugs with respective NOAC. The zero-point is without addition.

The dose-response characteristics of the PTr20/5 method for dabigatran, rivaroxaban, and apixaban run on the Simple Simon™ instrument showed a linear relationship for spiked plasma samples in comparison with the method at the central laboratory. Pearson’s correlation coefficients (*r*) between drug concentration and spiked plasma samples were 0.98, 0.99, and 0.99, respectively (Figure 1).

### The 5 μL, 10 μL, and 20 μL method, PTr20/5, and reference methods in authentic samples

Authentic samples with dabigatran, rivaroxaban, and apixaban were analyzed by the 20 µL, 10 µL, and the 5 µL modification of the study test, and the PTr20/5 was calculated for each sample.

Analysis of PT on Simple Simon™ using a 20 µL sample volume showed a linear correlation with the reference methods (for dabigatran *r* = 0.88, for rivaroxaban *r* = 0.92, and apixaban *r* = 0.89). Using a 10 µL sample volume also showed a linear correlation (for dabigatran *r* = 0.76, for rivaroxaban *r* = 0.91, and apixaban *r* = 0.78), and this was also the result using a 5 µL sample volume (for dabigatran *r* = 0.51, for rivaroxaban *r* = 0.86, and apixaban *r* = 0.19) (Supplementary Figure 2A–C).

**Figure 2.**
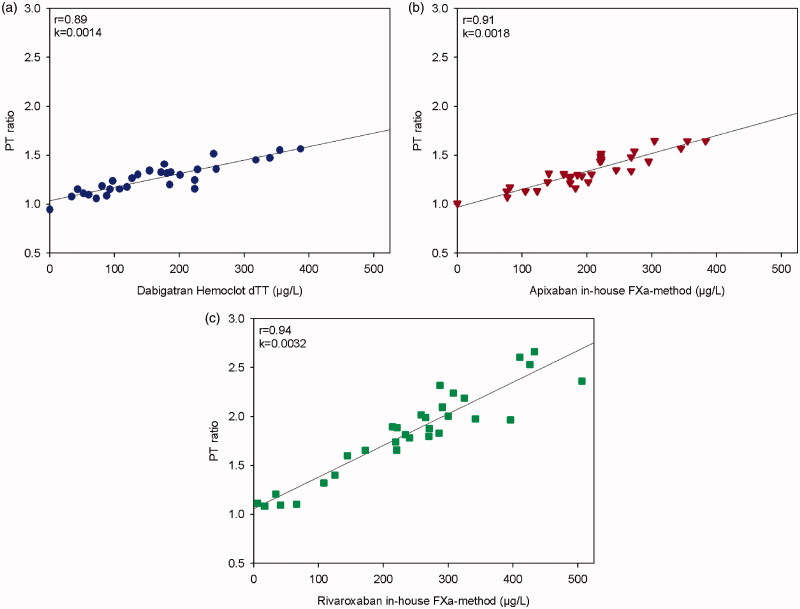
The dose-response characteristics of the PTr method run at room temperature on Simple Simon™ for dabigatran in comparison with Hemoclot dTT^®^ (a), and apixaban (b) and rivaroxaban (c) in comparison with our in-house anti-FXa methods. Plasmas from patients on treatment with the respective NOAC were used (*n* = 30); only one plasma sample from each patient. The zero-point is healthy donors without treatment.

The correlation for patient samples expressed as PTr20/5 with the reference method is shown in Figure 2(a–c), and *r* was 0.89, 0.94, and 0.91 for dabigatran, rivaroxaban, and apixaban, respectively. All correlations were statistically significant, *p* < 0.0001.

**Figure 3.**
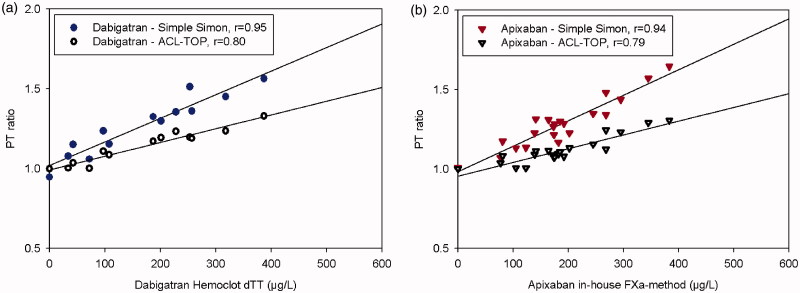
The dose-response characteristics of the PTr method run at +37 °C on ACL Top for dabigatran in comparison with Hemoclot™ dTT (a), and apixaban our in-house anti-FXa methods (b). Plasmas from patients on treatment with the respective NOAC were used. The zero-point is healthy donors without treatment. *n* = 12 in (a), *n* = 20 in (b).

Correlations for patient sample results obtained with the PTr20/5 procedure run at +37 °C on ACL Top compared with the central lab method were 0.97 (*p* < 0.0001) and 0.79 (*p* < 0.0001), respectively (Figure 3(a,b)).

According to the reference methods used in the central laboratory, the ranges of NOAC in the patient samples were from 40 µg/L to 400 µg/L for dabigatran, from 10 µg/L to 500 µg/L for rivaroxaban, and from 70 µg/L to 400 µg/L for apixaban.

### Temperature dependency of PT and PTr20/5 results

**Figure 4.**
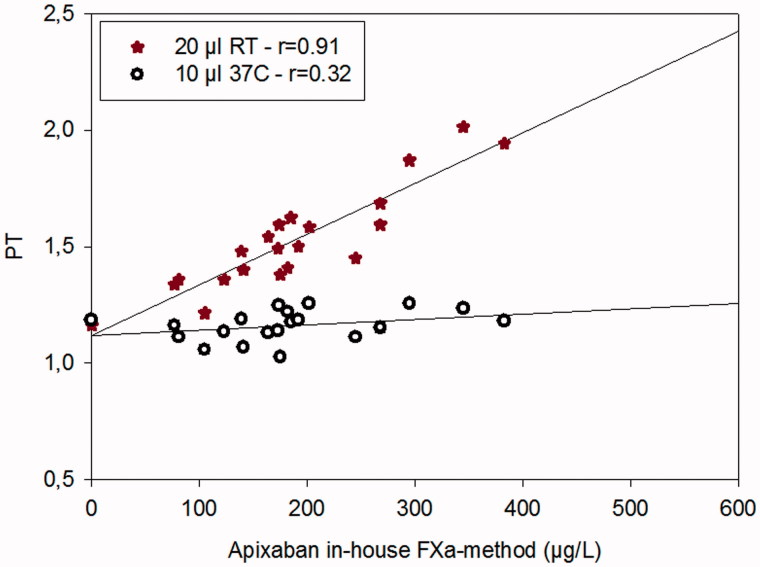
The dose-response characteristics of the PT method run at room temperature on Simple Simon™ for apixaban and PT measured on ACL Top at 37 °C in comparison with our in-house anti-FXa method run at +37 °C on ACL Top. *n* = 20. The zero-point is healthy donors without treatment.

In order to show the effect of temperature and the different PT reagents, two methods (the method run in the central lab and the Simple Simon method) were used to analyze plasma samples from patients on treatment with apixaban. The PT method used in the central lab uses a 10 µL sample volume and was run at 37 °C; Simple Simon™ uses same sample volume but was run at 22 °C (Figure 4).

The results of the PTr20/5 method run at room temperature ranged from 1.1 to 1.6 for dabigatran, from 1.1 to 2.7 for rivaroxaban, and from 1.1 to 1.7 for apixaban. The results of the PTr20/5 method run at +37 °C ranged from 1.1 to 1.7 for dabigatran and from 0.9 to 1.6 for apixaban (detailed data not shown).

### Sensitivity and precision measurements

To determine the sensitivity of the PTr20/5 method, 30 plasmas from normal individuals were analyzed at room temperature. The average PTr20/5 for normal plasmas was 1.02 and the SD was 0.06. From this it is concluded that a PTr20/5 of 0.12 (2 SD) is needed to distinguish an assay response from zero when only one determination of PT with 20 µL and one with 5 µL is performed. When we apply this variation to the results for patient samples, the limits of detection are thus 58 µg/L, 19 µg/L, and 81 µg/L for dabigatran, rivaroxaban, and apixaban, respectively.

The precision of the PTr20/5 method was estimated by determining the assay response 10 times for one patient plasma. The repeatability at this level was characterized by a CV of 2.94% for dabigatran (224 µg/L), 2.23% for rivaroxaban (240 µg/L), and 3.72% for apixaban (222 µg/L).

### Whole-blood measurements

The largest volume of sample, 20 µL, was chosen to also allow for blood analysis with the modified POC system studied. As a first check of the feasibility of whole-blood NOAC determination, three citrated blood samples from patients on rivaroxaban were analyzed by the 20 µL and 5 µL methods. The PTr20/5 for the three patient blood samples were 1.70, 1.32, and 1.73. In parallel analysis of the corresponding plasmas, these PT ratios were 1.92, 1.50, and 2.31, respectively. The corresponding rivaroxaban levels obtained from the plasma samples at the central lab were 367 µg/L, 236 µg/L, and 423 µg/L, respectively. It thus appears that analysis of whole blood by the studied method is possible, but that the sensitivity of blood analysis is reduced by about 35% compared to plasma analysis. In congruence to this, in the present small comparison between blood and plasma analysis, the detection limit for blood was estimated, about 63 µg/L, and as the steepness of curves is less for dabigatran and apixaban whole-blood determinations are not feasible at present for these drugs.

## Discussion

We have developed a new assay of NOAC in plasma, an ambient-temperature, wet-chemistry, prothrombin time (PT) procedure of the Owren-type utilizing the POC instrument Simple Simon™. The INR calibration procedure was originally intended only for monitoring of treatment with vitamin K antagonists, but is in reality used also when prothrombin time is analyzed in other conditions, for example as a risk marker for bleeding and liver disease. In general, the clinical laboratories have no information on whether plasma samples are from patients on vitamin K antagonist or not. In this study, we have expressed the PT results as INR for all samples. Certainly, an increased INR due to NOAC cannot be used to estimate bleeding risk.

In this method two or more PT determinations are made at different final dilutions of the sample, and the PT result, expressed as INR, is viewed only as a measure of the NOAC concentration. The rationale of this is that anticoagulants, and other inhibitors such as lupus anticoagulants (6), affect PT in a dose-dependent fashion, but decreasingly so as the final concentration of the plasma sample in the reaction mixture decreases. For example, PT Quick (plain thromboplastin), with a final plasma dilution of 1:3, is more strongly affected by NOACs than PT Owren (combined thromboplastin), with a final plasma dilution of 1:21, as reported previously (7–9). Furthermore, as shown in this study, the effect of NOACs is more prominent at +22 °C than at +37 °C.

Surprisingly, in the present study, the Owren PT modification, the PT 20 µL method, was at least as sensitive to apixaban as to dabigatran. As expected from previous reports (7–9) the 10 µL method, and even more so the 5 µL method, was insensitive to apixaban and dabigatran. Doubling the conventional sample volume for PT, i.e. 10 µL, resulted in more than twice as high an increase in response for apixaban. A further increase would probably lead to an enhanced response, although analysis of fresh capillary blood with optical read-out seems troublesome.

We chose to use the ratio of PT results obtained with the 20 µL and 5 µL samples. The ratio calculated, PTr20/5, made the method less influenced by the variation of INR the patients would have in the absence of NOACs and thus better correlated to the drug concentration. Thus, it may be possible to estimate the concentration of NOAC if basal INR is elevated due to deficiency in vitamin K or liver disease and even if the patient is on dual treatment with warfarin and a NOAC when switching treatment. Results would be only slightly improved if the value for zero addition of sample (i.e. infinite dilution and thus no effect of interfering substances) were to be extrapolated and used instead of the 5 µL value (not shown). Lowering the temperature to +22 °C effected a doubling of the response, and the combination of doubling the sample volume and decreasing the temperature gave an 11-fold increase in response for apixaban and similar results for rivaroxaban and dabigatran. Thus, a favorable temperature effect and the doubling of the sample volume enable the use of the modified PT Owren method to measure all NOACs using the same method and instrument. The PT method, which is run at ambient temperature on Simple Simon™ in its usual routine use, may be calibrated for all NOACs. The user only needs to know which novel anticoagulant to suspect in the sample. If it is known that the patient is treated with one of the NOACs but not which one, alternative estimated concentrations can be calculated. In a situation where it is not known if the patient is on anticoagulant treatment, the PTr20/5 might give a clue, and also run out higher levels of NOACs, VKAs, and heparins, even though it will not be possible to exclude lower (or, for some drugs, intermediate) concentrations of anticoagulants. This may be very useful also in conjunction with laboratory investigations of thrombophilia to avoid drawing erroneous conclusions due to analytical interferences (9). In this last-mentioned context, we believe present limits of detection of the PTr method run on Simple Simon™ are feasible.

It seems possible to measure rivaroxaban in fresh blood using the same approach, but for apixaban and dabigatran further development is needed to increase the ratio and thus the detection limit, sensitivity, and precision. This is also the case for applications on instruments run at 37 °C.

In conclusion, this novel modified PT method promises to be of use with dabigatran, rivaroxaban, and apixaban, and most likely also with other NOACs and perhaps also with other coagulation inhibitors. There are thus indications of a possible universal anticoagulant assay based on the cheap and most common coagulation assay, i.e. the prothrombin time, utilizing only one point-of-care instrument and one reagent.

## Supplementary Material

Supplemental data
