# Supplementary material for: A novel prothrombin time method to measure all non-vitamin K-dependent oral anticoagulants (NOACs)
Source: Ups J Med Sci. 2017 Sep 11;122(3):171–6. doi: 10.1080/03009734.2017.1370040 (PMC5649322; doi:10.1080/03009734.2017.1370040)
Supplement: Supplemental data [file IUPS_A_1370040_SM8517.docx]

# Supplemental section


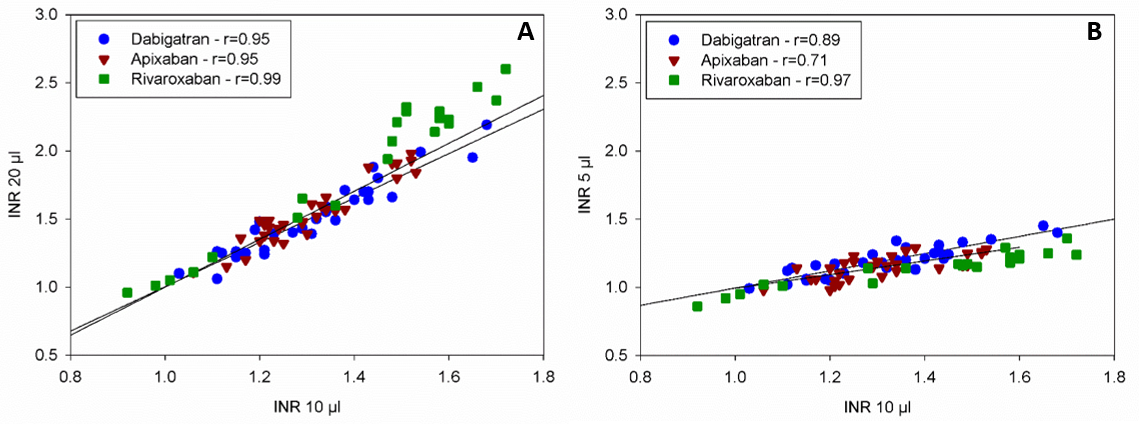


Supplemental Figure 1. The alignment between the results with 20 µL of sample and 10 µL of sample (Figure S-1A) and that of 5 µL of sample and 10 µL of sample (Figure S1-B).


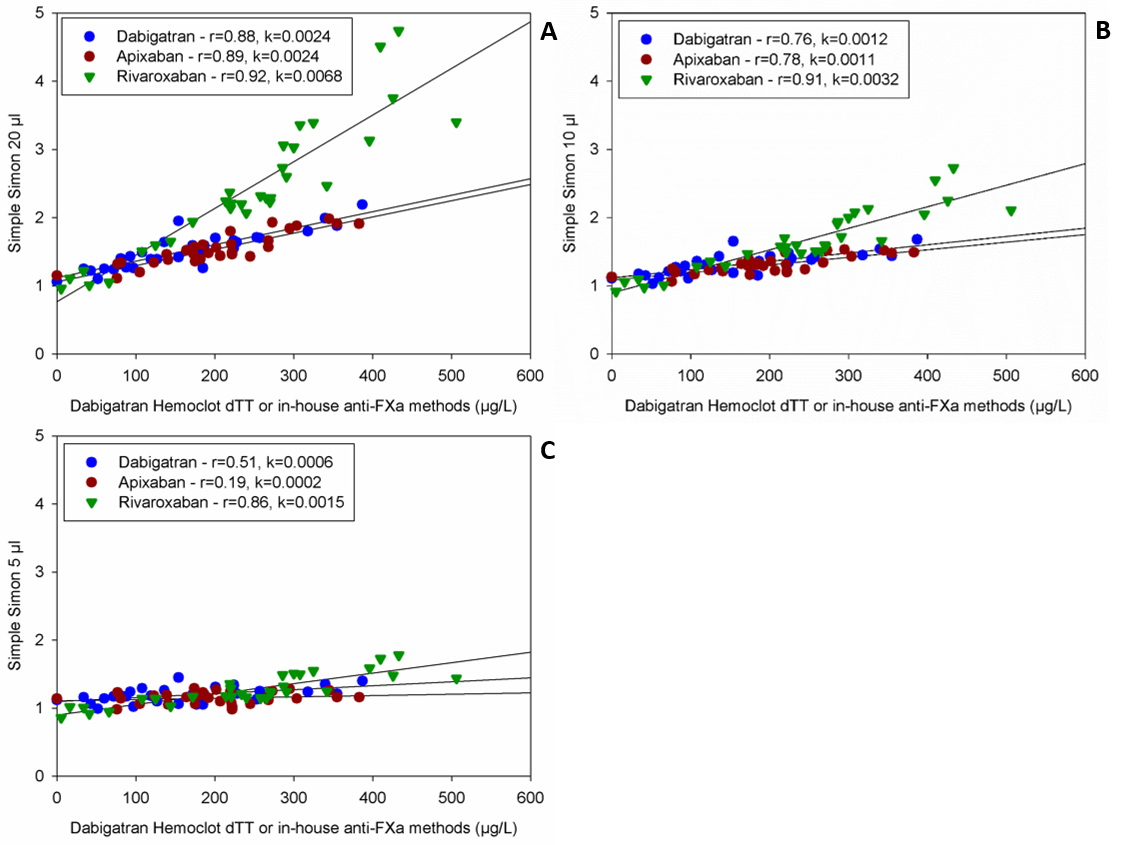


Supplemental Figure 2. The dose-response characteristics of the PT-method method variants run at room temperature on Simple Simon™ for 20 µL method (A), 10 µL method (B) and 5 µL method (C) in comparison with Hemoclot dTT^®^, or in-house anti-FXa methods, measured on plasmas from patients on treatment with respective NOAC. n=30. The zero-point is heathy donors without treatment
